# Recyclable Multilayer Packaging by Means of Thermoreversibly Crosslinking Adhesive in the Context of Food Law

**DOI:** 10.3390/polym12122988

**Published:** 2020-12-15

**Authors:** Katharina M. A. Kaiser, Johann Ewender, Frank Welle

**Affiliations:** 1 TUM School of Life Sciences Weihenstephan, Technical University of Munich, Weihenstephaner Steig 22, 85354 Freising, Germany; 2 Fraunhofer Institute for Process Engineering and Packaging IVV, Giggenhauser Strasse 35, 85354 Freising, Germany; johann.ewender@ivv.fraunhofer.de (J.E.); frank.welle@ivv.fraunhofer.de (F.W.)

**Keywords:** multilayer packaging, recycling, adhesive, Diels-Alder, EVOH, barrier testing, migration, diffusion coefficient, activation energy

## Abstract

Lacking recyclability of multilayer packaging can be overcome by using a thermoreversible crosslinking adhesive consisting of maleimide- and furan-functionalized polyurethane-(PU-)prepolymers, reacting in a Diels–Alder-reaction. Here, the furan-functionalized PU-prepolymer carries furan-side-chains to avoid the usage of an additional crosslinking agent. Thus, *N*‑(2‑hydroxyethyl)maleimide and furfurylamine are the only two chemicals contained in the adhesive that are not listed in the appendix of EU Regulation 10/2011. Using migration modelling, it could be shown that, at 23 °C, both chemicals have lag-times of only a few minutes if 45 µm PE is used as a barrier. However, if the residual content is below 30 mg/kg, the legally specified maximum amount of 0.01 mg/kg food is not reached. After determining the diffusion coefficients and the activation energy of diffusion through ethylene-vinyl alcohol copolymer (EVOH), it could be determined that the lag-time of the migrants can be extended to at least 9 years by the use of 3 µm EVOH. From a food law point of view, the use of the described adhesive is possible if the above‑mentioned measures are complied.

## 1. Introduction

In multilayer packaging, different materials are combined with each other in a layered structure, which enables the creation of customized property profiles. By combining the materials, defined barriers against oxygen, water vapor, light, or aroma can be created, thereby enabling extended shelf lives. Additionally, material combination provides the possibility to produce sealable packaging, achieve certain optical effects, and obtain desired mechanical properties with low material consumption. Because of these advantages, multilayer packaging is an important part of the packaging market [1,2,3,4].

However, these multilayered films are usually not recyclable, as many different materials are incompatible and thus cannot be processed together [5,6,7]. According to the European Plastics Strategy, all packaging should be reusable or recyclable by 2030 [8]. To achieve this goal, innovations in design-for-recycling and recycling methods must therefore be promoted.

In a previously published article [9], the approach of producing multilayer packaging with thermoreversibly crosslinking adhesives and recycling them with a corresponding solvent-based recycling process was presented. PET-PE, PE-aluminum, and PET-aluminum laminates were produced using an adhesive containing Diels–Alder adducts. Under the influence of temperature in DMSO, the laminates can be delaminated by inducing the retro-Diels-Alder reaction. The individual film materials (PE, PET, aluminum) can be recovered without adhesive residues and thus made accessible for recycling [9,10]. In post-consumer recycling, such packaging could therefore be recovered after it has been sorted out of the post-consumer packaging stream using appropriate technologies. The technologies that can be envisaged for such sorting include built-in marcers based on fluorescence, photoluminescence, or digital watermarks, which enable identification and sorting. [11,12,13,14,15]. One option for sorting multilayer packaging without markers is based on the fact that multilayer films usually curl up when exposed to temperature and can therefore be separated by wind sifting [16].

The problem with the adhesive system described in the previous paper is that the carcinogenic chemical furfuryl alcohol [17] and a toxicologically unevaluated cross-linker, a molecule with three furan groups, were used. However, in food packaging, it is also important that all substances contained in the adhesive system are safe and that all requirements under food law are met. According to EU Regulation 10/2011, chemicals included in the Union list of authorized substances may be used in food contact materials without further regulation [18]. Non-listed chemicals that are carcinogenic, mutagenic, or toxic to reproduction may not be used without prior authorization. However, non-listed chemicals that are not carcinogenic, mutagenic, or toxic to reproduction may be used behind a functional barrier, provided that a maximum migration limit of 0.01 mg/kg into the food is respected [18].

According to the method described in the previous paper, *N*-(2-hydroxyethyl)maleimide, furfuryl alcohol, and a crosslinker molecule with three furan groups were used in addition to common packaging adhesive components [9,19]. While *N*-(2-hydroxyethyl)maleimide is allowed to be used as a non‑cancerogenic/reprotoxic/mutagenic (CRM) chemical behind a functional barrier, furfuryl alcohol is potentially carcinogenic and the cross-linker molecule is not toxicologically evaluated because it is not commercially available. These two chemicals should therefore not be used in food packaging without prior testing and approval by the European Food Safety Authority.

Accordingly, the adhesive formulation in this paper contains, in addition to common components of packaging adhesives, only furfurylamine and *N*-(2-hydroxyethyl)maleimide. These two chemicals may both be used behind functional barriers, provided the maximum migration limit of non-listed substances behind a functional barrier is respected [20,21]. Since crosslinking of the adhesive is ensured by furfuryl side chains on the furfuryl-prepolymer, the use of an additional crosslinking molecule is unnecessary.

The aim of the study is to discuss the modified adhesive formulation with regard to its suitability for packaging and recycling purposes, which on the one hand means that sufficient adhesive strength and good separability of the packaging has to be achieved. Additionally, for food law evaluation, the migration behavior of furfurylamine and *N*‑(2‑hydroxyethyl)maleimide through PE, PE/EVOH/PE, PET has to be assessed. For this purpose, the diffusion coefficients of the migrants through PET, ethylene–vinyl alcohol copolymer (EVOH) and activation energies of diffusion have to be determined. Based on the experimentally determined activation energies of diffusion and the residue contents of free furfurylamine and *N*-(2-hydroxyethyl)maleimide it can be determined how much of the migrants can migrate into the packaged food at the end of shelf life.

On this basis it will be discussed under which conditions the adhesive could be used for food packaging from a food law perspective.

## 2. Materials and Methods

### 2.1. Characterization by Infrared Spectroscopy

The Fourier transform infrared (FTIR) spectrometer from Perkin Elmer (Shelton, CT, USA) and the corresponding software Spektrum One were used to measure and process IR spectra. The measurements were performed with an ATR (Golden Gate, Perkin Elmer, Shelton, CT, USA) measurement unit, whereby ten scans were carried out for each spectrum. The measured wavelength range was 4000 to 600 cm^−1^.

### 2.2. Determination of the Molecular Weight

The size exclusion chromatography was performed in THF. For this purpose, 50 mg of each sample was dissolved in 10 mL THF and 40 µL of this solution was then injected using an ASI-100 Automate Sample Injector Unit from Dionex Corporation (Sunnyvale, CA, USA). For calibration, a narrow molecular weight distribution polystyrene standard (PSS Polymer Standards Service, Mainz, Germany) was used. The measurements were performed with a flow rate of 1 mL∙min^−1^ (Bischoff HPLC Compact Pump) at 40 °C. The refractive index detector RID-6A from Shimadzu (Kyoto, Japan) was selected as the detector. The separation column used was the GPC/SEC-column SDV, linear M, 300 × 8 mm, 5 µm, 100 Å of PSS. Evaluation of the elution curves was performed with the PL Cirrus GPC/SEC Software (Version 1.2).

### 2.3. Preparation of the Adhesive

4,4′-Methylene diphenyl diisocyanate (MDI) (98%) was obtained from Alfa Aesar (Ward Hill, MA, USA) and used as received, if a dimer formation could be excluded. Furfurylamine (≥99%, Merck, Darmstadt, Germany), the polyesterdiol Capa™ 2054 (Perstorp, Malmö, Sweden) was dried prior to use at reduced pressure and under a slightly elevated temperature. In addition, an isocyanate prepolymer from Morchem SA (Les Franqueses del Vallès, Spain) was used for the preparation of the furan-functionalized prepolymer.

The maleimide functional group, *N*-(2-hydroxyethyl)maleimide, was prepared via a Diels–Alder and retro-Diels–Alder reaction in a three-step process [22,23]. Methyl ethyl ketone (≥99.5%) was purchased from TH Geyer and, if necessary, dried with molecular sieve (4 Å) to an H_2_O content <50 mg/kg and stored under nitrogen (H_2_O ≤ 50 mg/kg). Reactions involving the use of isocyanate compounds were carried out under a nitrogen atmosphere.

### 2.4. Furan-Functionalized Prepolymer

**Figure 1:**
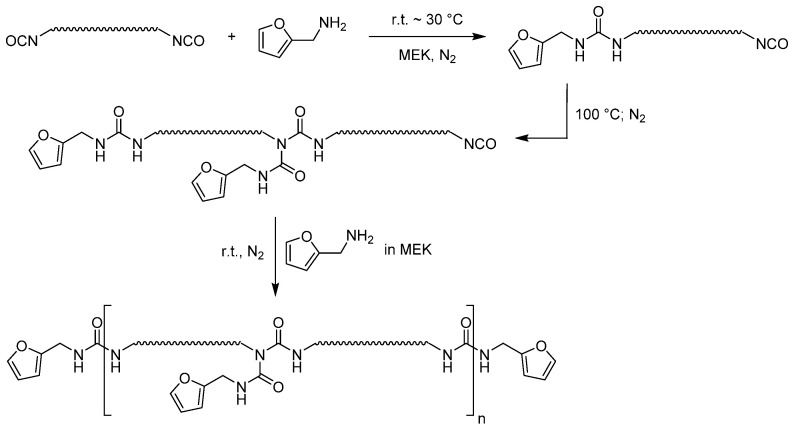
Reaction scheme for the preparation of the furan prepolymer with side groups.

To produce the furan-functionalized prepolymer (see Figure 1, Figure S1), the isocyanate prepolymer (152 g, solids content 106 g, 43.2 mmol, 3.00 equiv.) was added to a three-necked flask, which was set under nitrogen and equipped with a mechanical stirrer. Afterwards, 2.80 g (28.8 mmol, 2.67 mL, 1.00 equiv.) furfurylamine were diluted with methyl ethyl ketone (MEK) and were slowly added to the stirred prepolymer so that the temperature inside the flask would not exceed 30 °C. The reaction mixture was then stirred for 30 min at room temperature, before heating the oil bath to 110 °C. It could be observed that the reaction mixture initially became clear and then turned yellowish with time. The mixture was stirred at this temperature until the isocyanate signal at 2264 cm^−1^ in the IR did not decrease any further. It can therefore be assumed that all urea groups have reacted with isocyanate groups. Then, the mixture was cooled to about 70 °C and 20 mL MEK were added to keep the polymer stirrable. To finish the reaction, 2.80 g (28.8 mmol, 2.67 mL, 1.00 equiv.) furfurylamine in 10 mL MEK was then added to this viscous polymer solution to functionalize the remaining NCO-groups.

The product was obtained as a clear, yellowish viscous polymer solution with a solids content of 83%.

Details on material characterization can be found in the Supporting Information.

This polymerization was carried out similarly to the method of Du et al. [24].

### 2.5. Maleimide-Functionalized Prepolymer

**Figure 2:**
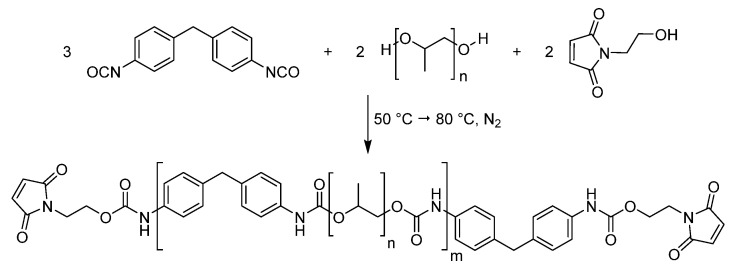
Reaction scheme for the preparation of the maleimide-prepolymer.

To produce the maleimide-functionalized prepolymer (see Figure 2, Figure S2), MDI (4.50 g, 18 mmol, 3.00 equiv.) was heated to 50 °C to give a clear, colorless liquid to which 12.0 g (12 mmol, 2.00 equiv.) polypropylene glycol 1000 was added all at once. After the exothermic reaction subsided, the mixture was heated to 80 °C until reaction control with FTIR showed no further decrease of the NCO-signal. Subsequently, 1.69 g (12 mmol, 2.00 equiv.) *N*-(2-hydroxyethyl)maleimide was added to the mixture. The reaction was kept at 80 °C until the NCO-signal vanished.

The polymer was obtained as a clear, yellow viscous and tacky polymer.

Details on material characterization can be found in the Supporting Information.

### 2.6. Preparation of the Adhesive Mixture

**Figure 3:**
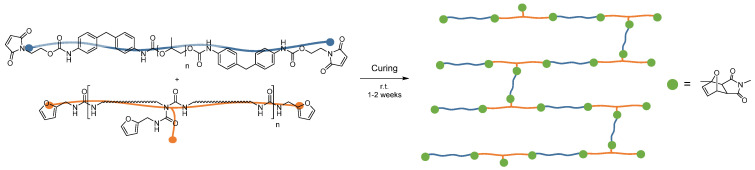
Cross-linking reaction of the two adhesive components during the curing process.

To prepare the adhesive (see Figure 3), total of 0.78 g (52.0 mmol of maleimide-groups, 1.00 equiv.) of the maleimide-prepolymer was weighed into a vial together with 1.20 g (52.0 mmol of furan-groups, solids content 1.00 g) of the furan polymer and dissolved in 4.15 g MEK by shaking. In this way, a low-viscous, pale yellow solution with 30% solids content was obtained.

Details of the infrared spectrum can be found in the Supporting Information.

### 2.7. Production of Laminates

Before use, the polymer films were treated with corona (PET: 600 Watt/5 m/min, PE: 1000 Watt/5 m/min, surface tension >38 mN/m) and then cut into pieces in DIN A4 format. The coating was applied by means of the CUF 5 (Sumet Messtechnik, Denklingen, Germany) coating unit at an application speed of 40 mm/s, a drying temperature of 70 °C, and a drying time of 60 s. To achieve the target dry film thickness of 8 µm, a wired rod with 27 µm wet film application was used. The films used were PET (23 µm, Hostaphan), PE (45 µm, Hanita), and aluminum foil (20 µm). For curing, the samples were stored at room temperature under the pressure of a 5 kg weight.

The thickness of the coating was checked using a precision thickness gauge FT3 (Rhopoint Instruments, Beyhill on Sea, UK) with an accuracy of 0.1 µm at five randomly selected positions on the film with a repeatability accuracy of 0.5 µm. The values were averaged for closer examination. However, since the film thicknesses of PE, PET, and aluminum foils also appeared to fluctuate, the adhesive thickness was also partially checked by microtome sections. Microtome sections were prepared using the device Jung Autocut 2055 of Leica Microsystems GmbH (Wetzlar, Germany). The samples were each cut into microtomes with thickness of 20 µm and analyzed using an optical microscope with objectives of 40- and 200-times magnification.

### 2.8. T-Peel Test

A Schenk-Trebel universal testing machine type RM 50 from Bischoff Prüftechnik GmbH (Solingen, Germany) was used for tensile testing. Before the measurement, the laminates were stored for at least 24 h at 23 °C and 50% relative humidity and cut into 15-mm wide strips. The laminates were tested perpendicular to the direction of lamination. During the entire measurement, there was an angle of 90° between the measuring direction and the non-separated part of the sample. The measuring speed was 50 mm/min in all cases and the measuring range was 60 mm, while the measuring range between 5 and 60 mm was used for the analysis. The Test & Motion program from Doli Elektronik GmbH (Münsingen, Germany) was used for evaluation.

### 2.9. Statistical Hypothesis Testing

Five bond strength values were determined per laminate and then subjected to statistical analysis to determine the significant differences in bond strength between laminate types, using Visual-XSel 12.0 Multivar (CRGRAPH, Munich, Germany). For this purpose, the five values for each laminate were first examined for normal distribution using the Anderson-Darling normality test, with a significance value of 0.05. A *t*-test was used to compare two laminates since a normal distribution of the five samples was present in all laminates.

### 2.10. Recovery of the Materials

**Figure 4:**
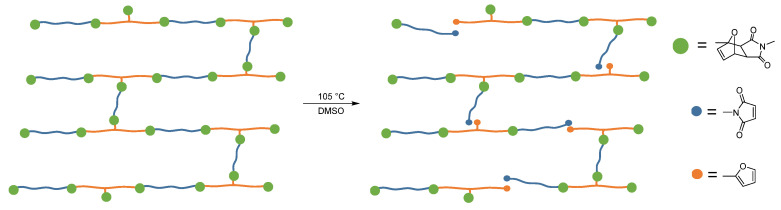
The retro-Diels–Alder reaction opens the polymer network sufficiently to solvate the polymer fragments.

With scissors, the laminates were each cut into 20 square pieces with dimensions of 1 × 1 cm and then added to 50 mL DMSO. Resulting mixture was then heated to 105 °C and stirred with a stirring fish at a speed of 400 rpm (compare Figure 4). The delamination process was monitored both by observation and by occasional sampling.

### 2.11. Determination of Residual N-(2-hydroxyethyl)maleimide and Furfurylamine

The unreacted amount of furfurylamine and *N*-(2-hydroxyethyl)maleimide was determined using gas chromatography–mass spectrometry (GC-MS). Since a slightly different residual content of maleimide or furan is expected in each polymerization batch, two batches per prepolymer were analyzed. This allows an estimation of how well the residual amount of furfurylamine and *N*‑(2‑hydroxyethyl)maleimide can be controlled.

The analyses were carried out using a Trace GC 1310 unit from Thermo Fisher Scientific Inc. (Waltham, MA, USA) with a liquid autosampler and mass spectrometry coupling. The separation column used was an Rxi-624 Sil-MS, with a length of 30 m, an inner diameter of 0.25 mm, and a layer thickness of 1.4 µm. After an isothermal time of 2 min at 50 °C, the temperature was raised up to 320 °C at a rate of 10 °C/min and kept at this temperature for 15 min. A Thermo DSQII, quadrupole MS unit in electronic ionization mode was used as the MS-unit. The measuring method was selective ion monitoring and the measured fragment masses for furfurylamine were *m*/*z* 69, 77, 80, 86 and for *N*-(2-hydroxyethyl)maleimide m/z 82, 98, 110, 111. The quantification was performed for each substance using an external standard series. The detection limit determined for *N*‑(2‑hydroxyethyl)maleimide was 10–15 mg/kg, for furfurylamine of 10 mg/kg.

In this way, it was determined that the residual content of furfurylamine in both furan-functionalized prepolymers was approximately 15.0 mg/kg (compare Table 1). The residual content of maleimide in the two maleimide-functionalized prepolymers was quite different, which can be attributed to slightly different amounts weighed during synthesis. Therefore, calculations were performed for both maleimide concentrations in the following discussion.

### 2.12. Determination of the Ethylene Content in the Measured EVOH Barrier

DSC was performed on a DSC 821e instrument of Mettler-Toledo GmbH (Gießen, Germany) following the DIN EN ISO 11357-1 method. Samples were heated from 23 °C to 200 °C at a heating rate of 10 K∙min^−1^. Two heating runs were performed with 6–10 mg of sample. The program used for evaluation was Mettler-Toledo’s STARe software, whereas the second heating run was used for evaluation.

The analysis showed that the EVOH used has a melting point of 183 °C, implying that the ethylene content is 32%.

### 2.13. Determination of Diffusion Coefficients in EVOH

Diffusion coefficients of organic substances in EVOH were determined from the lag times of the permeants through a PE/EVOH/PE film, with an EVOH thickness of 3 µm and PE layers of 25 µm and 30 µm respectively. For this purpose, the EVOH films were clamped in a permeation steal cell between two sealant rings. The surface area of the tested films was 191 cm^2^. The permeation cell was placed in a climate chamber, that has a lower and an upper space separated by the film, while the lower space of the permeation cell had a volume of 7667 cm^3^. The permeants of different polarities and functional groups (overall 38, listed in Tables S7–S11) are injected as a liquid mixture into the lower space of the permeation cell through a septum by use of a syringe. After injection, the liquid mixture of permeants evaporated immediately at the high temperatures (65 °C to 80 °C) applied in the permeation tests. The upper space of the permeation cell was permanently rinsed with a pure stream of nitrogen (20 mL/min) which moved the permeated substances out of the cell. The nitrogen stream went through a connected enrichment unit and the permeants were trapped on this unit over a period of 20 min. The enrichment unit was connected to a gas chromatograph with flame ionization detection (GC/FID). The permeants were directly desorbed into the gas chromatograph and the amount of permeants was determined quantitatively. During the GC run, the next sample was trapped on the enrichment unit and subsequently injected into the GC. By use of this method, one kinetic point was measured every 45 min. Gas chromatographic conditions: column: Rxi 624, length: 60 m, internal diameter: 0.32 mm, film thickness: 1.8 µm, carrier gas: 100 kPa helium. Temperature program: 40 °C (2 min), rate 10 °C/min to 200 °C, rate 20 °C/min to 260 °C hold for 5 min. Pre-trap: substances collected on 20 mm length by 5 mm diameter of Carbopack B, desorbed at 300 °C. Main trap: substances focused at −46 °C on 20 mm length by 1.4 mm diameter of Carbopack B, desorbed at 320 °C. Calibration was performed with injections of known amounts of the applied permeants. The diffusion coefficients (*D_P_*) were determined according to the lag time method [25,26,27] (Equation (1)) and the activation energies of diffusion (*E_A_*) were calculated from the *D_P_* at various temperatures according to the Arrhenius Equation (Equation (2)).

(1) $$lag\;time=\frac{l^{2}}{6D_{P}}$$

*D_P_* is the diffusion coefficient of the permeant in the barrier film, *l* is the thickness of the film.

(2) $$D_{P}=D_{0}\;e^{-\frac{E_{A}}{RT}}$$

*D_0_* is the pre-exponential factor, *R* is the gas constant and *T* is the temperature in Kelvin.

### 2.14. Diffusion Modelling

For diffusion modelling, the AKTS SML software version 4.54 (AKTS AG Siders, Sierre, Switzerland) using finite element analysis was used. The mathematical principles on which the program is based were published by Rodiut et al. [28].

The packaging geometry for which the calculations were carried out was chosen in accordance with the commission regulation (EU) No 10/2011 with a contact surface between package and food of 6 dm^2^ and a food volume of 1 kg.

Since the furan- and maleimide-functionalized prepolymers are mixed in a two-component adhesive, the concentrations of furfurylamine and *N*-(2-hydroxyethyl)maleimide contained in the adhesive are reduced. With the ratio of the two polymers used in the present case, 9 mg/kg residual content of furfurylamine and 14 mg/kg/47 mg/kg residual content of *N*-(2-hydroxyethyl)maleimide were obtained in the adhesive used. These values were used for the migration modeling as initial concentrations of the migrants in the adhesive.

Material-specific settings that have been selected to model the migration for the PET‑PE/EVOH/PE laminate are given in Table 2 and for PET-PE/EVOH/PE laminate in Table 3. The diffusion coefficients used can be found in Table 4 and reflect the scenario at 23 °C.

Since the density of the PU adhesive is not known, a density of 1.10 g/cm^3^ was assumed, which is about an average value for unfoamed PU materials. [29] For the medium air, the very high diffusion coefficient of 0.02 cm^2^/s was assumed since this value reflects that the volatile migration substances are directly transported away from the surface. For PE, the diffusion coefficient according to Piringer at 23 °C was selected, since determination according to Piringer was found sufficient [30]. The modelling parameters for PU were not given in the scientific literature. Therefore, the diffusion coefficients valid for PE were also selected for the PU adhesive, which can be considered as the worst case for PU. In this way, a fast diffusion of the migrant out of the adhesive was assumed and thus, a worst-case scenario is represented. Also, to represent a worst-case scenario, the distribution coefficients were chosen as 1 in all cases.

## 3. Results and Discussion

### 3.1. Adhesion and Recyclability

**Figure 5:**
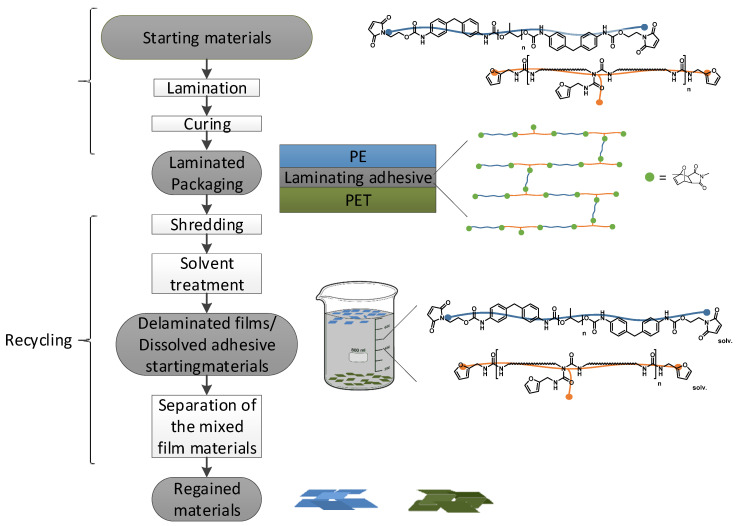
Schematic presentation of the complete laminate production and recycling process: PET-PE, PET-aluminum, and PE‑aluminum laminates were produced with an adhesive made of maleimide/furan-functionalized PU prepolymers, with the furan prepolymer carrying furan side chains for crosslinking. For subsequent recycling, the laminates must first be shredded and then treated with a heated solvent to cause the retro-Diels–Alder reaction and then the dissolution of the adhesive components. The separation of PE from PET and aluminum was achieved by skimming the PE from the surface of the solvent. Adapted from [9].

An overview of the entire process, which consists of laminate production using Diels-Alders adhesive and the subsequent recycling of the laminate, is shown in Figure 5.

The formulation of the adhesive was chosen in such a way that crosslinking of the adhesive is achieved without the need for an additional crosslinking molecule, since the furan-functionalized prepolymer contains furan-functionalized side chains. The ratio between the starting materials of the furan-functionalized prepolymer was chosen such that on average three functional groups are contained in the polymer (see material & methods). In many formulations, functionalization with *N*‑(2‑hydroxyethyl)maleimide results in the presence of solid prepolymers. This is not necessarily an obstacle to successful bonding, but it does prevent the adhesive from being tacky during the lamination process, which can cause problems because of the stresses that are applied to the laminates in the laminating machine and during winding. Therefore, polypropylene glycol was used in the maleimide-functionalized prepolymer as a diol, as it ensures that this prepolymer is a viscous liquid at room temperature, thereby creating tack in the lamination process and allowing the adhesive to flow better onto the surface of the laminated film.

Furthermore, the formulation was designed to allow dissolution of furan- and maleimide-prepolymers in MEK. This was especially important for the furan-functionalized prepolymer, as the urea and biuret groups contained in the prepolymer provided a low solubility, probably due to hard segment phases formed by the urea-groups [32]. MEK is suitable as a solvent for solvent-based adhesives for packaging purposes because it is non-toxic and has a low boiling point, making it a common solvent for these purposes [33].

**Figure 6:**
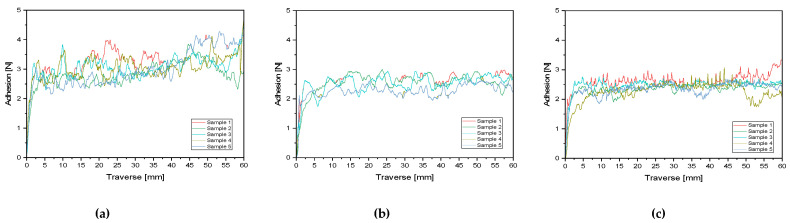
Results of the T-Peel test of the three laminate types (**a**) PET-PE, (**b**) PET-aluminum, and (**c**) PE-aluminum.

The bond strength results achieved with this adhesive system on PE-PET, PE-aluminum, and PET‑aluminum laminates are between 2 and 3 N/15 mm and are shown in Figure 6 and Table 5. Details on the measurements are given in Tables S1–S6. The significance test showed that the bond strength in the PET‑PE laminate was significantly different than in the PET‑aluminum and PE‑aluminum laminates. The two aluminum laminates, however, did not differ significantly in their bond strength. On closer inspection of the laminates separated by performing the T-Peel Test, it can be seen that in the case of the aluminum laminates, an adhesion failure occurred, whereas in both cases, the adhesive adhered to the plastic film and no residues on the aluminum film can be detected. In the case of the PET‑PE laminates, however, a cohesive failure was observed. As the adhesion to aluminum seems to be weaker than to PET and PE, the lower forces in the case of the PET‑aluminum and PE‑aluminum laminates can be explained. One possible explanation for the lower adhesion to aluminum could be, that typically no entanglement is possible between adhesive and metal surfaces. [34,35] The measured bond strengths were supposed to be sufficient for packaging in most cases.

After the bonding of the laminates could be proven by T-Peel test, the laminates were recycled in a laboratory scale. The laminate flakes cut to 1 cm^2^ were treated at 105 °C in DMSO. The times determined for delamination of the laminates, without further mechanical impact, are shown in Table 6.

Within 40 min, which is still a reasonable time for recycling processes, all laminates were separated from each other. However, the delamination speed could be influenced by a smaller film piece size, as this increases the surface area of the cut edges through which the solvent can penetrate the adhesive. Similarly in the previous work, [9] the PE-containing laminates PE-PET and PE‑aluminum are separated faster than the PET-aluminum laminate, since the diffusion coefficient of PET is significantly higher than that of PE [36], especially since the melting point of PE is almost reached at 105 °C.

In the case of the two PE laminates PET‑PE and PE‑aluminum, the PE can be skimmed off the surface of the solvent because of density differences, while PET and aluminum, on the other hand, sink to the ground. An eddy current separator would be necessary to separate PET and aluminum in a mixed fraction. After removal of the solvent adhering to the surface of the flakes by exposure to temperature at reduced pressure, HS-GC was used to determine 17 mg/kg solvent in PE and 9224 mg/kg in PET. These would need to be removed by a solvent degassing during re-extrusion if the process were to be carried out on a larger scale. A more detailed description of the proposed upscaled version of the process can be found in the previous publication [9]. Furthermore, no residues of the adhesive polymers can be detected on the films by means of infrared spectrometry (see Figures S3 and S4).

### 3.2. Migration Modelling of N-(2-hydroxyethyl)maleimide and Furfurylamine through PE According to Piringer

With the exception of *N*-(2-hydroxyethyl)maleimide and furfurylamine, all chemicals used in the adhesive formulation are listed in the appendix of the EU Regulation 10/2011. As these two chemicals are not carcinogenic, mutagenic, or toxic for reproduction, the regulation states that their use in an intermediate layer of the packaging is acceptable, provided that it can be ensured by using a functional barrier that less than 0.01 mg of the substance is released into 1 kg of the packed goods. As described in the Material and Methods section, 121 mg/kg and 36 mg/kg residual *N*‑(2‑hydroxyethyl)maleimide were determined in the maleimide polymer. For the furfuryl functionalized prepolymer, about 15 mg/kg residual content of furfurylamine was repeatedly determined. Since the two polymers were mixed in the form of a two-component adhesive, the concentrations of furfurylamine and *N*-(2-hydroxyethyl)maleimide were reduced. With the ratio of the two polymers used in the present case, 9 mg/kg residual content of furfurylamine and 14 mg/kg/47 mg/kg residual content of *N*-(2-hydroxyethyl)maleimide were thus present in the adhesive. The following migration predictions were carried out with these concentrations as initial concentrations in the adhesive layer.

**Figure 7:**
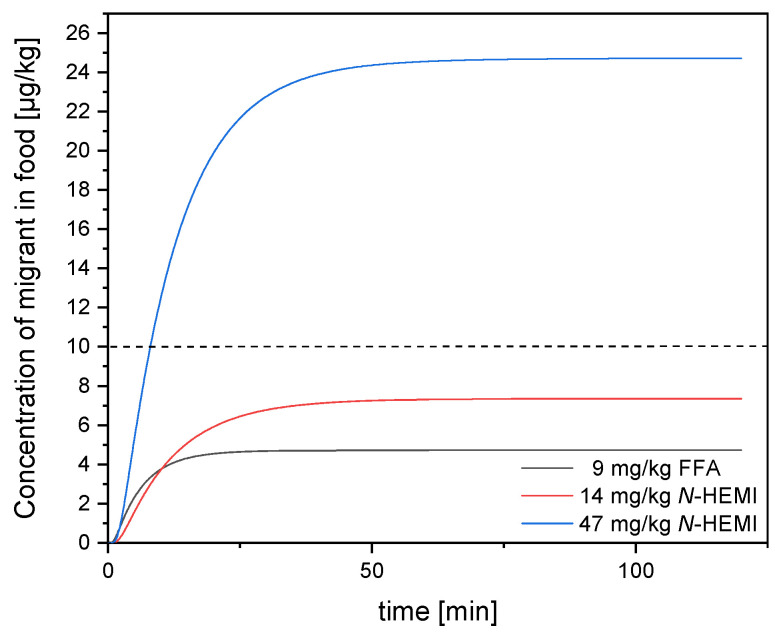
Visualization of the time-dependent concentration of migrants in food using a PE barrier of 45 µm at 23 °C over 120 min. The concentration curves are shown for a residual content of 9 mg/kg furfurylamine (black) and 14 mg/kg and 47 mg/kg NHEMI (red and blue) in the adhesive. The dotted line indicates the legally prescribed limit value.

In the case of a PET‑PE laminate, which is very common on the packaging market, it can be assumed that the PE side of the laminate faces the filling material, as this is the sealable side. Therefore, the following section investigates how the migration of the residual contents of furfurylamine and *N*‑(2‑hydroxyethyl)maleimide of the adhesive through a PE film in a PET‑PE-laminate is to be assessed. Assuming that in such a package a 45-µm thick PE film is the only barrier to the contents, the migration scenarios for furfurylamine and *N*-(2-hydroxyethyl)maleimide shown in Figure 7 could be modelled. In the case of furfurylamine, the lag time, i.e., the time after which the first molecules have migrated into the food at 23 °C, is 95 s, while in the case of *N*-(2-hydroxyethyl)maleimide, lag time is 187 s. For furfurylamine, the final concentration was set after 25 min with an amount of 0.005 mg furfurylamine per kg of foodstuff. For *N*‑(2‑hydroxyethyl)maleimide, the final concentration was reached after 40/50 min with 0.007 mg/kg *N*‑(2 hydroxyethyl)maleimide in the best case and 0.025 mg/kg in the worst case.

Thus, the migrants penetrated the PE barrier to food almost immediately, which shows that PE does not act as a suitable barrier against the migrants. Even if the thickness of the PE was increased from 45 µm to 1000 µm, i.e., more than 22 times as thick, the lag-time is 13 h for furfurylamine and 26 h for *N*‑(2‑hydroxyethyl)maleimide. Thus, even such unrealistically thick PE layers do not represent a sufficient barrier for the lifetime of most packaging purposes.

**Figure 8:**
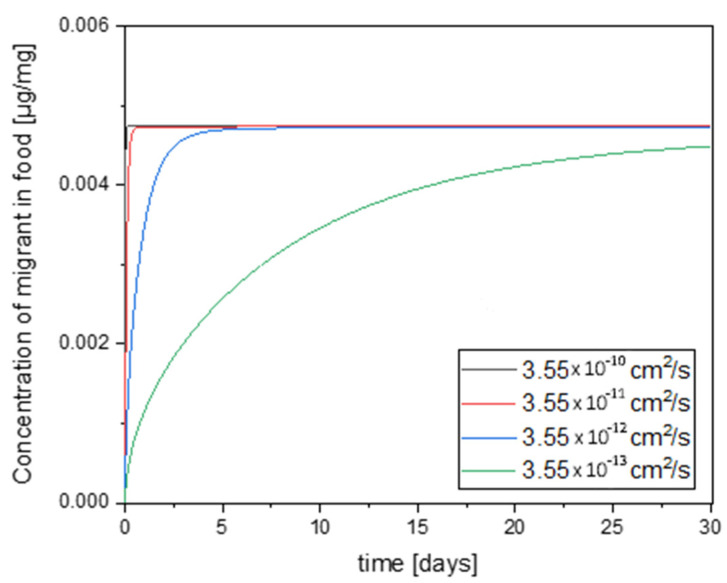
Time-dependent increase in concentration of furfurylamine in food in a PET-PE laminate using different diffusion coefficients of the migrant in the adhesive at 23 °C.

The speed at which the migrant passes into the food also depends on the speed at which it migrates from the adhesive to the PE layer. This speed is influenced by the diffusion coefficient of the migrant in the adhesive. The previous simulations were carried out, as indicated under Material and Methods, under the assumption that the migrant in the adhesive has the same diffusion coefficient as in PE. With this assumption, a very high mobility of *N*-(2-hydroxyethyl)maleimide and furfurylamine is observed in the adhesive and thus a fast transition into the neighboring polymer layers is assumed. This assumption creates a worst-case scenario, but a slower transition would be realistic because it can be assumed that the free volume in the adhesive is lower than in PE because of the stronger intermolecular interactions and crosslinking and because of relatively high structural affinity of the migrants to the adhesive system [37]. To assess the effect of this worst-case estimation, the order of magnitude of the diffusion coefficient of PE was gradually adjusted to the magnitude of the diffusion coefficient in PET. Figure 8 shows the time-dependent concentration of the migrant in the food under this variation of the diffusion coefficient in the adhesive in a PET-PE laminate.

The lag time remains the same if the diffusion coefficient in the adhesive is varied, as this has little influence on the molecules that come directly from the boundary layer. However, the supply of migrants is slowed down, which means that the maximum concentration in the food is reached more slowly. If the diffusion coefficient in the adhesive was 3.60 × 10^−12^ cm^2^/s, the maximum concentration would be reached after about 2 days, while diffusion coefficient of 3.60 × 10^−13^ cm^2^/s will cause the final concentration to be reached after about 25 days.

The actual diffusion coefficient for furfurylamine should be between the assumed values 3.60 × 10^−9^ cm^2^/s and 3.60 × 10^−13^ cm^2^/s. The exact value would have to be determined separately, but is probably irrelevant in the present situation, as the maximum concentration is also reached within one day at 3.60 × 10^−10^ cm^2^/s and 3.60 × 10^−11^ cm2/s. Should the diffusion coefficient of furfurylamine be in the order of 3.60 × 10^−12^ cm^2^/s and 3.60 × 10^−13^ cm^2^/s, a possibly relevant time delay could occur.

Figure 7 showed that despite the rapid lag-time at room temperature, the maximum amount of furfurylamine that can pass into the foodstuff is 0.005 mg/kg at an initial concentration of 9 mg/kg in the adhesive. In the case where a residual content of 14 mg/kg *N*‑(2‑hydroxyethyl)maleimide is present in the adhesive, the maximum concentration that can be transferred to the food in the current packaging layout is 0.007 mg/kg, which is also below the limit value. The situation is different with a residual content of 47 mg/kg *N*‑(2‑hydroxyethyl)maleimide in the adhesive. Here, the amount that can migrate into food is 0.025 mg/kg and the limit value would therefore be exceeded. For furfurylamine and *N*‑(2‑hydroxyethyl)maleimide it could be determined that in the present packaging design the limit value is not exceeded if the residual content of *N*‑(2‑hydroxyethyl)maleimide and furfurylamine in the adhesive is lower than 30 mg/kg.

Accordingly, the synthesis conditions of the adhesive and quality controls must guarantee that this threshold is not exceeded. The same also applies to the multilayer structure PET‑aluminum‑PE, since the adhesive directly contacts the PE layer facing the food.

Since migrants pass through PE so quickly into the food, the barrier effect of EVOH on migrants is examined below. For this purpose, it is first of all necessary to determine the diffusion coefficients of the migrants and structurally related molecules through EVOH, since the use of the diffusion coefficients determined according to Piringer would result in an overestimation of the values in this case.

### 3.3. Diffusion Coefficients

**Figure 9:**
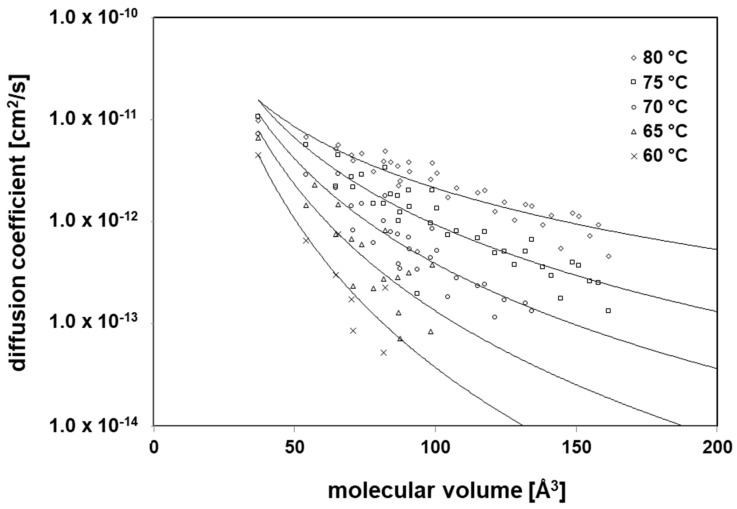
Correlation between the molecular volume (*V*) of the permeants and their diffusion coefficients (*D_P_*) in EVOH films at various temperatures.

The diffusion coefficients were determined from the lag times of the applied permeants through the PE/EVOH/PE film according to Equation (1). Due to the fact that EVOH is a low diffusive polymer, temperatures of 61 °C up to 81 °C have to be applied. At lower temperatures, the diffusion through the PE/EVOH/PE film was too slow and the permeated amounts were below the analytical detection limits if any of the molecules was able to overcome the barrier. Permeation through the PE layer at such high temperatures is much faster, which means that the lag times of the PE layers are negligible under the applied temperature conditions. Therefore, the experimentally determined diffusion coefficients are related to the EVOH layer. The applied temperatures are above the *T*_g_ of EVOH. Below *T_g_*, diffusion in glassy state is lower which means that the experimentally determined diffusion coefficients can be considered as a worst-case. The experimentally determined *D_P_* values for the applied 38 substances are given in Tables S7–S11 and are shown in Figure 9, where it can be seen that the tested substances show correlations between their molecular volume (*V*) and their diffusion coefficients *D_P_*. Similar correlation has been found in previous studies on the same method [25,26,27]. The applied substances followed the Arrhenius relationship (Equation 2) from which the activation energies of diffusion were derived for 15 of the 38 applied substances. The other substances follow the same relationship. However, the temperature range was too small or the amount of diffusion coefficients was less than four kinetic points, which was the minimum amount of individual diffusion coefficients for the calculation of the activation energies. These strict rules are necessary. Otherwise, the activation energies of diffusion will show too high uncertainty. The activation energies of diffusion are given in Table S12 in the Supporting Information.

### 3.4. Activation Energies of Diffusion

**Figure 10:**
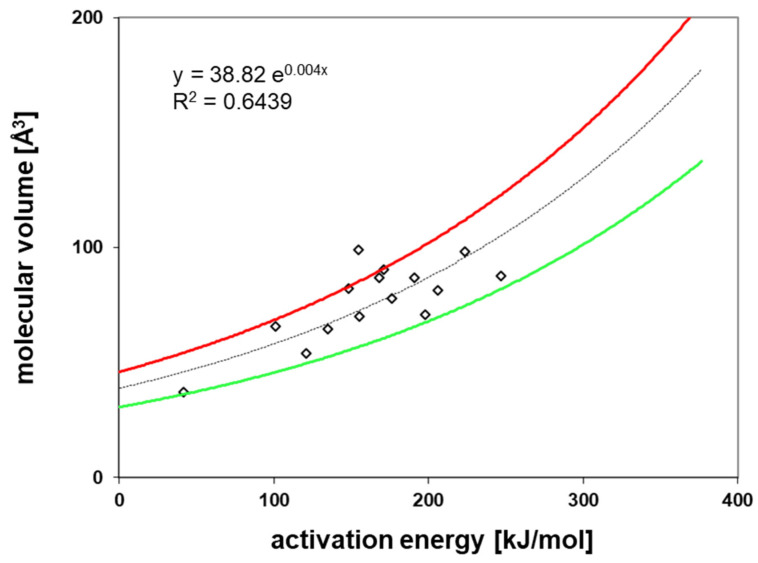
Correlation between the activation energy of diffusion (*E_A_*) and the molecular volume (*V*) of the test permeants.

**Figure 11:**
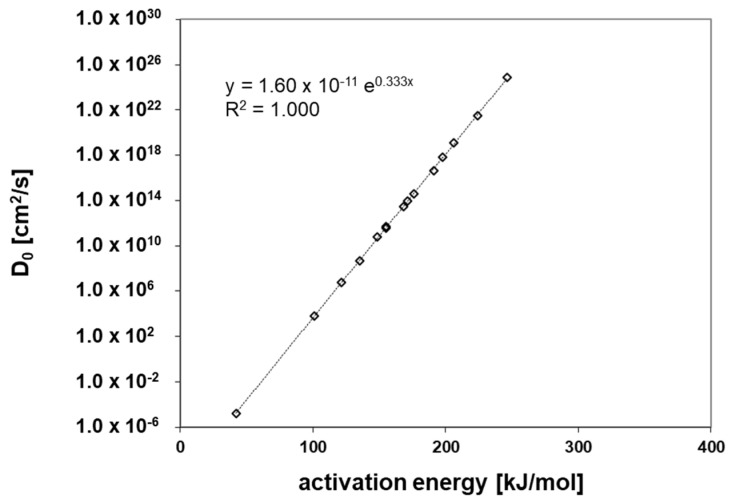
Correlation between the activation energy of diffusion (*E_A_*) and the pre-exponential factor (*D_0_*) of the test permeants.

*E_A_* and the *D_0_* values for each permeant were calculated from the Arrhenius relationship (Equation 2). The results show good linearity for the investigated permeants, which indicates that the diffusion process follows Fick’s laws of diffusion, and that the swelling of the polymer by the permeants can be excluded under the experimental conditions in this study. For *E_A_* and *D_0_* the results are given in Table S13. Figure 10 and Figure 11 show a correlation of the *E_A_* to the *V* and the *D_0_* of the investigated permeants, respectively. From both correlations, the slope and the intercept of the correlations were derived which are the basis of the prediction of the diffusion coefficients at low temperatures, e.g., 23 °C is the temperature applied in the diffusion modelling (see below). The prediction of the diffusion coefficients is based on the method published by Welle [31] and based on Equation (3).

(3) $$D_{P}\;=\;b\;\left(\frac{V}{c}\right){\;}^{\frac{a\;-\;\frac{1}{T}}{d}}$$

where *D_P_* is the diffusion coefficient (in cm^2^/s), *V* is the molecular volume (in Å^3^), *T* is the temperature (in K), and the parameters *a* to *d* the slope and the intercepts of the above correlations between *V* and *E_A_* and *D_0_* versus *E_A_* with a = 2.77 × 10^−3^ 1/K, b = 1.60 × 10^−11^ cm2/s, c = 38.82 Å^3^), and d = 3.36 × 10^−5^ 1/K.

### 3.5. Migration Modelling of N-(2-hydroxyethyl)maleimide and Furfurylamine through EVOH

The diffusion coefficients predicted for furfurylamine and *N*-(2-hydroxyethyl)maleimide for PET [31] and EVOH (this study, from Equation (1)) at 23 °C were used to discuss the migration through PE/EVOH/PE-barrier instead of PE, facing the packed food. We have to predict the diffusion coefficients of both molecules, because experimentally the diffusion coefficients were available only at temperatures of 60 °C and above. This is an indication, that both PET and EVOH are good barrier polymers for organic substances.

**Figure 12:**
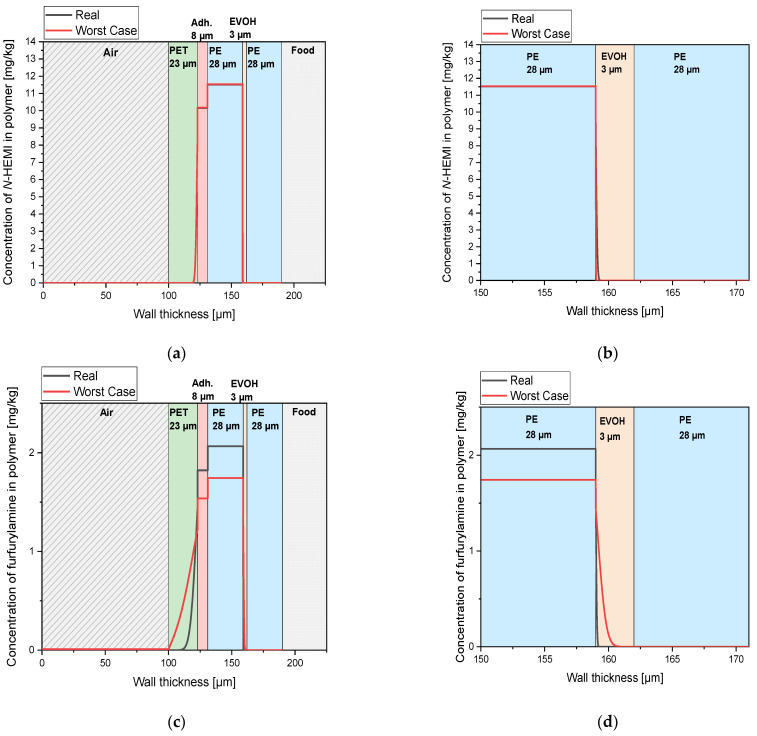
Concentration profile of migrants in the film structure PET‑PE/EVOH/PE after one year at 23 °C. (**a**) Concentration profile for the migrant *N*-(2-hydroxyethyl)maleimide (*N*-HEMI), both in a worst-case and in a realistic scenario. For better recognition of the barrier effect of EVOH, the area of the EVOH layer is shown enlarged in (**b**). (**c**) Worst-case and realistic concentration profile for furfurylamine. An enlarged representation of the barrier effect of EVOH is shown in (**d**).

Figure 12 depicts the concentration profiles of *N*-(2-hydroxyethyl)maleimide (13 a + b) and furfurylamine (13 c + d) in a PET‑PE/EVOH/PE laminate (see material and methods) after one year at 23 °C.

It can be seen that the concentration of migrants in the adhesive and the adjacent PE layer is high and decreases sharply from the interfaces with PET and EVOH. Since diffusion into PE is very fast, the migrants are distributed very quickly between the adhesive and the adjacent PE film. The higher concentrations in PE can be explained by the fact that the concentration is given in mg/kg and PE has a lower density than the adhesive. Since the diffusion coefficients for *N*-(2-hydroxyethyl)maleimide in PET and EVOH are so low, the *N*‑(2‑hydroxyethyl)maleimide migrates only 7.4 µm from the adhesive/PET interface into the PET and only 0.6 µm from the PE/EVOH interface into the EVOH within one year.

Because of the faster diffusion of furfurylamine, it penetrates further into PET and EVOH. Through the 23-µm thick PET layer, furfurylamine has a lag time of about one year according to the worst-case scenario, whereas the 3 µm EVOH barrier is not overcome after one year.

**Figure 13:**
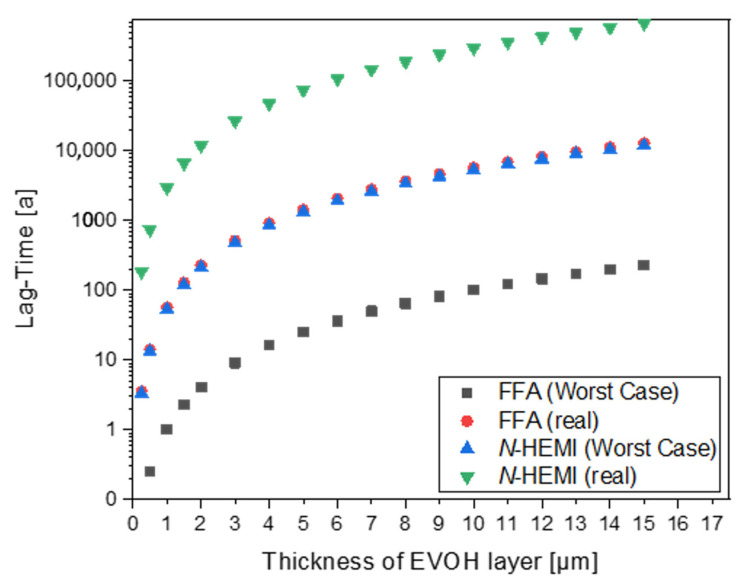
Dependence of the lag time of *N*-(2-hydroxyethyl)maleimide (*N*-HEMI) and furfurylamine on the layer thickness of EVOH at 23 °C.

Figure 13 shows the correlation between the thickness of the EVOH barrier and the lag times of the two migrants at 23 °C, where the layer thickness has, according to Equation 1., a quadratic effect on the lag time. Even in the worst-case scenario of furfurylamine, a 1.5-µm thick layer of EVOH ensures a lag time of 3 years, a reasonable time for most packaging purposes. However, such thin EVOH layers are not common, as they would be difficult to extrude [38]. Ordinary EVOH layer thicknesses start at around 3 µm, and would thus provide a lag time of 9.13 years (realistic scenario) or 513 years (worst case scenario) for furfurylamine. In the case of *N*-(2-hydroxyethyl)maleimide the lag times are 476 years (worst-case scenario) or even around 26,500 years in the realistic scenario. Since even the lowest breakthrough time of 9.13 years determined for furfurylamine in the worst-case scenario is more than sufficient for normal packaging purposes, EVOH is a suitable barrier for the adhesive.

However, in terms of recyclability, however, the use of PE/EVOH/PE compounds makes only limited sense. While low concentrations of EVOH have only a minor impact on the quality of the recycled material, PE-EVOH compounds with an EVOH content >5% are not considered recyclable [39,40]. Assuming that the thinnest possible extrudable EVOH layer is about 3 µm thick, the adjacent PE layers would have to be at least 30-µm thick each. However, in the case of a PET tray with a PE/EVOH/PE layer (e.g., used for products with antioxidant requirements) [41], where the PET could be made available for recycling by separating the PE-EVOH portion, it would also be a gain to recycle only the PET portion, as this has the decidedly higher mass portion. From this point of view, it would also be reasonable to use a PE/EVOH/PE film with an EVOH content >5%. In addition, the PE/EVOH/PE portion could be recycled by mixing it into a PE fraction to dilute the EVOH content.

## 4. Conclusions

Multilayer packaging fulfils an important function in the packaging market, but is under constant criticism because it usually cannot be recycled. However, they can be made recyclable by using a reversible crosslinking packaging adhesive with Diels-Alder-adducts, which would have to meet all legal requirements and must not endanger the safety of the consumer. With the formulation used in this work, for the laminates PET‑PE, PE‑aluminum, and PE‑aluminum bond strength between 2 and 3 N/15 mm could be achieved, which is supposed to be sufficient for most packaging purposes. The separation of the material components for recycling takes place in heated solvent, in which the Diels–Alder adducts open and the components are then solvated in the solvent.

For furfurylamine and *N*‑(2‑hydroxyethyl)maleimide, the two chemicals that are according to the EU Constitution 10/2011, not allowed to reach a concentration of 0.01 mg per kg of food, diffusion models had been used to predict the diffusion behavior of the multilayer packaging materials. Using the diffusion coefficients according to the Piringer model it can be shown that the lag-time of furfuryl alcohol and *N*‑(2‑hydroxyethyl)maleimide through 45-µm thick PE at 23 °C is only a few minutes. For packaging applications, therefore, this would not be considered a functional barrier against the two migrants. However, whether the limit value of 0.01 mg/kg per kg of foodstuff specified in the EU Regulation 10/2011 is exceeded depends on the residual content of the migrants in the foodstuff. Provided that the content of both substances in the adhesive is below 30 mg/kg the limit of 0.01 mg/kg in food is not exceeded.

To investigate, how an EVOH barrier can affect the migration of the two chemicals, the diffusion coefficients of the migrants through PET and EVOH and activation energies of diffusion had to be determined. Based on these data, the prediction of the diffusion coefficients of furfurylamine and *N*‑(2‑hydroxyethyl)maleimide (or any other organic migrant) was possible according to the Welle prediction model. It could be shown that EVOH acts as a good functional barrier and a 3-µm thick EVOH layer provides an effective barrier against migrants for food purposes.

In summary, it can be said that the modified adhesive formulation has the necessary bond strength and the principle of recyclability by means of the solvent-based recycling process is given. The migration modelling showed that the use of the adhesive behind an EVOH barrier should be possible without restrictions under food law. In order to be able to make statements about the feasibility of the lamination and the recycling process on a larger scale, experiments have to be carried out on a small pilot scale. These and experiments on the behavior of the adhesive during packaging processing will be covered in a future publication.

## Figures and Tables

**Table 1:** Residual content of furfurylamine and *N*-(2-hydroxyethyl)maleimide determined via GC-MS.

		Measurement 1	Measurement 2
**Residue of furfurylamine**			
Furan-functionalized prepolymer 1	mg/kg	15.9	15.0
Furan-functionalized prepolymer 2	mg/kg	15.3	13.5
**Residue of *N*-(2-hydroxyethyl)maleimide**			
Maleimide-functionalized prepolymer 1	mg/kg	36.0	35.5
Maleimide-functionalized prepolymer 2	mg/kg	120.6	122.0

**Table 2:** Material-specific settings used for the PET-PE-laminate.

		Air	PET	Adhesive	PE
**Thickness**	µm	100	23.0	8.00	45.0
**Density**	g/cm^3^	1.20 × 10^−3^	1.38	1.10	0.97
**Partition coefficient**		1.00	1.00	1.00	1.00

**Table 3:** Material-specific settings used for the PET-PE/EVOH/PE-laminate.

		Air	PET	Adhesive	PE	EVOH	PE
**Thickness**	µm	100	23.0	8.00	28.0	3.00	28.0
**Density**	g/cm^3^	1.20 × 10^−3^	1.38	1.10	0.97	1.16	0.97
**Partition coefficient**		1.00	1.00	1.00	1.00	1.00	1.00

**Table 4:** Diffusion coefficients used for diffusion modelling, if not stated otherwise.

Migrant	Layer	Scenario	Diffusion Coefficient 23 °C	Source
	Air	/	0.02 cm^2^/s (assumed as worst case)	
**Furfurylamine**	Adhesive	/	3.55 × 10^−8^ cm^2^/s	Diffusion coefficient assumed as for PE
	PE	/	3.55 × 10^−8^ cm^2^/s	Piringer [30]
	PET	real	2.78 × 10^−15^ cm^2^/s	[31]
		worst case	2.39 × 10^−14^ cm^2^/s	[31]
	EVOH	real	9.26 × 10^−19^ cm^2^/s	this study
		worst case	5.21 × 10^−17^ cm^2^/s	this study
***N*-(2-Hydroxyethyl) maleimide**	Adhesive	/	1.81 × 10^−8^ cm^2^/s	Diffusion coefficient assumed as for PE
	PE	/	1.81 × 10^−8^ cm^2^/s	Piringer [30]
	PET	real	2.25 × 10^−16^ cm^2^/s	[31]
		worst case	1.94 × 10^−15^ cm^2^/s	[31]
	EVOH	real	1.79 × 10^−20^ cm^2^/s	this study
		worst case	1.00 × 10^−18^ cm^2^/s	this study

**Table 5:** Mean values and standard deviations of the bond strength measurements related to the 15 mm width of the test strips. The numerical values indicated the mean value from five measurements. The corresponding measurement curves can be found in Figure 6.

Laminate		Mean Value
PET‑PE	N/15 mm	3.07 ± 0.11
PET‑aluminum	N/15 mm	2.30 ± 0.41
PE‑aluminum	N/15 mm	2.39 ± 0.33

**Table 6:** Delamination times of the laminates. Since not all flakes delaminate at the same time, the start time of the delamination and the end of the delamination are given.

Laminate	PET-PE	PE-Aluminum	PET-Aluminum
Start of delamination	27 min	29 min	35 min
End of delamination	33 min	36 min	40 min

## Supplementary Materials

The following are available online at https://www.mdpi.com/2073-4360/12/12/2988/s1. Figure S1. Chemical structure of the maleimide-prepolymer. Figure S2. Chemical Structure of the furan-prepolymer. Table S1. Adhesion strength of the 5 test specimens. F gives the average force and Fmax the maximum force of the sections selected for evaluation. Fa represents the measured initial force. Table S2. Mean values, standard deviations, and variation coefficients von F, Fmax, and Fa of the five test specimens. Table S3. Adhesion strength of the five test specimens. F gives the average force and Fmax the maximum force of the sections selected for evaluation. Fa represents the measured initial force. Table S4. Mean values, standard deviations, and variation coefficients von F, Fmax, and Fa of the five test specimens. Table S5. Adhesion strength of the five test specimens. F gives the average force and Fmax the maximum force of the sections selected for evaluation. Fa represents the measured initial force. Table S6. Mean values, standard deviations, and variation coefficients von F, Fmax, and Fa of the five test specimens. Figure S3. Spectra of the delaminated PET films and the untreated film. Figure S4. Spectra of the delaminated PE films and the untreated film. Table S7. Diffusion coefficients (DP) in EVOH for the homologous row of 1-alcohols. Table S8. Diffusion coefficients (DP) in EVOH for the homologous row of 2-ketones. Table S9. Diffusion coefficients (DP) in EVOH for oxygen-containing heterocycles. Table S10. Diffusion coefficients (DP) in EVOH for aromatic substances. Table S11. Diffusion coefficients (DP) in EVOH for the homologous row of formate esters determined from PE/EVOH/PE film. Table S12. Activation energies of diffusion EA and pre-exponential factors D_0_ determined within this study. Table S13. Prediction of diffusion coefficients of furfurylamine and *N*-(2-hydroxyethyl)maleimide.
