# Supplementary material for: Recyclable Multilayer Packaging by Means of Thermoreversibly Crosslinking Adhesive in the Context of Food Law
Source: Polymers (Basel). 2020 Dec 15;12(12):2988. doi: 10.3390/polym12122988 (PMC7765215; doi:10.3390/polym12122988)
Supplement: Supplementary file 1 [file polymers-12-02988-s001.pdf]

Supplementary Information

Recyclable multilayer packaging by means of thermoreversibly crosslinking adhesive in the context of food law

Katharina Kaiser[1,2 *], Johann Ewender[2], Frank Welle[2]

1. Technical University of Munich, TUM School of Life Sciences Weihenstephan, Weihenstephaner Steig 22, 85354 Freising, Germany;
2. Fraunhofer Institute for Process Engineering and Packaging IVV, Giggenhauser Strasse 35, 85354 Freising, Germany;

Table of Contents

1. Adhesion strength measurement 2

2. Evaluation of recovered materials by IR spectroscopy 6

3. Diffusion coefficients determined for the applied permeants. 8

4. Determined activation energies for some of the measured permeants. 10

5. Prediction of diffusion coefficients for *N*-(2-hydroxyethyl)maleimide and furfurylamine 11

1. Characterization of the Prepolymers.

Maleimide-Prepolymer

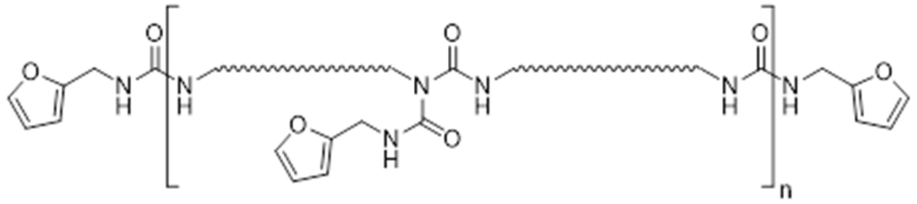

Figure S1. Chemical structure of the maleimide-prepolymer.

IR ṽ = 3315 (br), 2971 (m), 2937 (m), 2876 (m), 1713 (s), 1597 (m), 1531 (s), 1475 (w), 1460 (w), 1413 (m), 1372 (m), 1308 (m), 1219 (s), 1169 (m), 1066 (s), 1018 (m), 937 (w), 817 (w) 767 (w), 732 (w), 630 m).

**GPC**	Furan-functionalized prepolymer 1	Mw	51932 g/mol	PDI	3.85
	Furan-functionalized prepolymer 2		49911 g/mol		3.19

Furan-Prepolymer

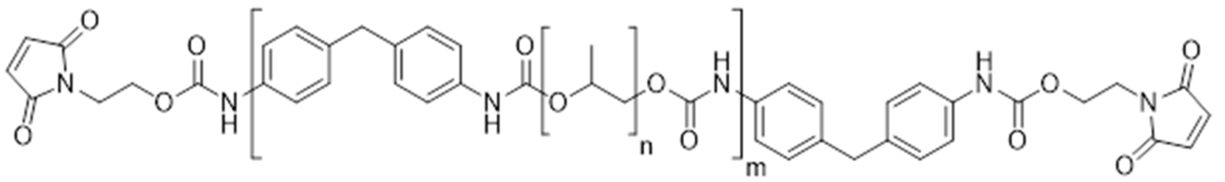

Figure S2. Chemical Structure of the furan-prepolymer.

IR ṽ = 3301 (br), 2971 (m), 2868 (m), 1711 (s), 1598 (m), 1531 (s), 1451 (w), 1411 (m), 1373 (m), 1343 (w), 1309 (m), 1221 (s), 1076 (vs), 1017 (s), 926 (m), 821 (m), 768 (w), 696 (m).

**GPC**	Maleimide-functionalized prepolymer 1	Mw	13921 g/mol	PDI	2.08
	Maleimide-functionalized prepolymer 2		15002 g/mol		2.21

Cured Adhesive

IR ṽ = 3299 (br), 2971 (m), 2872 (m), 1724 (s), 1702 (s), 1598 (m), 1532 (s), 1458 (w), 1413 (m), 1374 (m), 1342 (w), 1310 (m), 1222 (s), 1072 (vs), 1018 (s), 930 (m), 853 (s), 817 (m), 767 (m), 730 (m), 716 (m).

2. Adhesion strength measurement

PET-PE

Table S1. Adhesion strength of the 5 test specimens. F gives the average force and Fmax the maximum force of the sections selected for evaluation. Fa represents the measured initial force.

	F	Fmax	Fa
	[N]	[N]	[N]
Sample 1	3.20	4.16	3.10
Sample 2	3.10	3.84	3.21
Sample 3	2.90	3.85	2.91
Sample 4	3.10	4.12	3.31
Sample 5	3.06	4.28	3.04

Table S2. Mean values, standard deviations and variation coefficients von F, Fmax and Fa of the 5 test specimens.

	F	Fmax	Fa
	[N]	[N]	[N]
Mean value	3.07	4.05	3.11
Standard deviation	0.11	0.20	0.15
Variation coefficient	3.64	4.84	4.92

PET-aluminium

Table S3. Adhesion strength of the 5 test specimens. F gives the average force and Fmax the maximum force of the sections selected for evaluation. Fa represents the measured initial force.

	F	Fmax	Fa
	[N]	[N]	[N]
Sample 1	2.44	2.98	2.02
Sample 2	2.63	3.00	2.70
Sample 3	2.05	2.82	1.95
Sample 4	2.06	2.75	2.16
Sample 5	2.32	2.69	2.14

Table S4. Mean values, standard deviations and variation coefficients von F, Fmax and Fa of the 5 test specimens.

	F	Fmax	Fa
	[N]	[N]	[N]
Mean value	2.30	2.85	2.02
Standard deviation	0.25	0.14	0.30
Variation coefficient	10.8	4.83	13.5

PE-aluminium

Table S5. Adhesion strength of the 5 test specimens. F gives the average force and Fmax the maximum force of the sections selected for evaluation. Fa represents the measured initial force.

	F	Fmax	Fa
	[N]	[N]	[N]
Sample 1	2.62	3.12	2.03
Sample 2	2.38	2.67	2.45
Sample 3	2.50	2.77	2.45
Sample 4	2.48	3.10	2.27
Sample 5	2.38	2.69	2.32

Table S6. Mean values, standard deviations and variation coefficients von F, Fmax and Fa of the 5 test specimens.

	F	Fmax	Fa
	[N]	[N]	[N]
Mean value	2.47	2.87	2.30
Standard deviation	0.10	0.22	0.17
Variation coefficient	4.03	7.75	7.49

3. Evaluation of recovered materials by IR spectroscopy

The spectra of both film sides of the recovered materials and the spectrum of a virgin, untreated film plotted on top of each other.

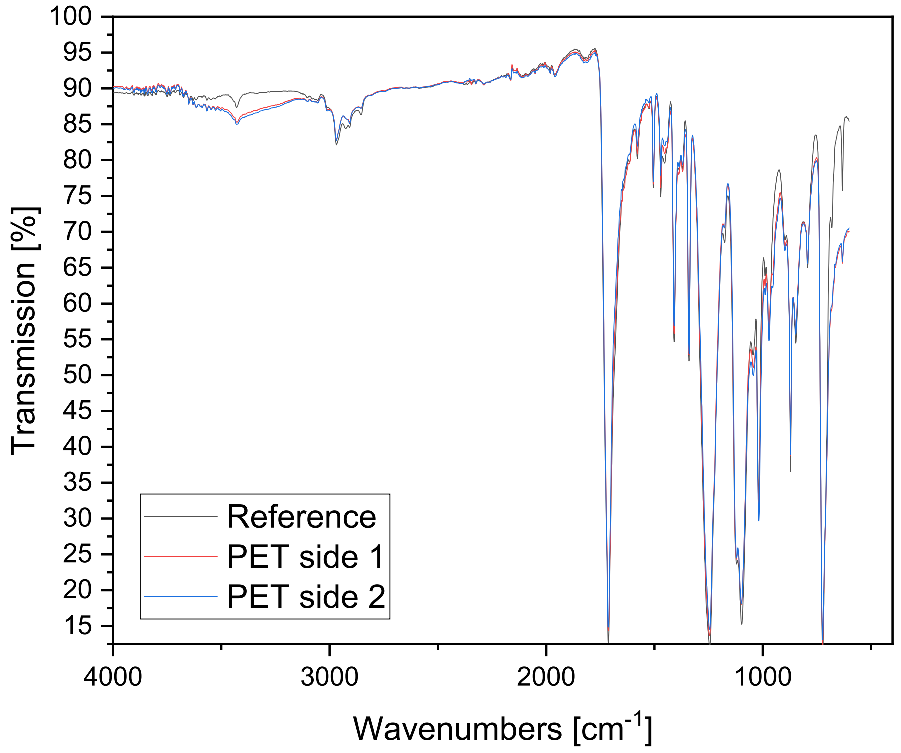

Figure S3. Spectra of the delaminated PET films and the untreated film.

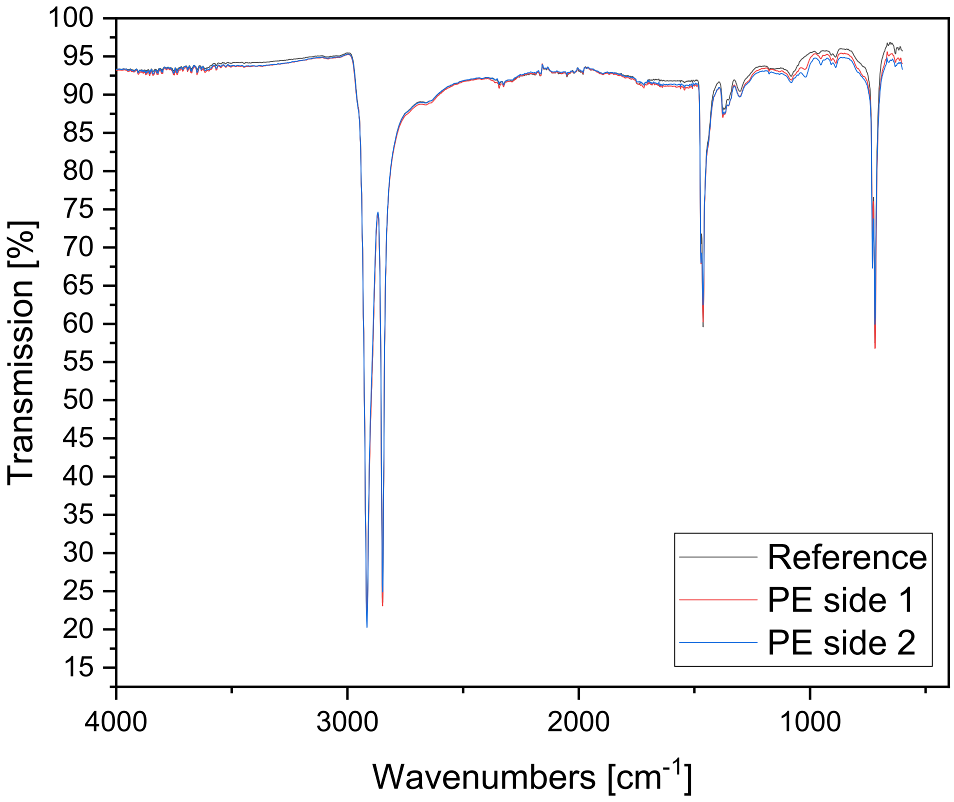

Figure S4. Spectra of the delaminated PE films and the untreated film.

4. Diffusion coefficients of measured substances

Table S7. Diffusion coefficients (D_P) in EVOH for the homologeous row of 1-alcohols.

	Molecular volume	Molecular weight	D_P				
	Å^2	g/mol	cm^2/s				
			81.3 °C	76.0 °C	71.0 °C	66.9 °C	61.3 °C
methanol	37.21	32.0	9.92 10^{-12}	1.08 10^{-11}	7.27 10^{-12}	6.66 10^{-12}	4.48 10^{-12}
ethanol	54.02	46.1	6.84 10^{-12}	5.66 10^{-12}	2.90 10^{-12}	1.46 10^{-12}	6.56 10^{-13}
1-propanol	70.82	60.1	3.96 10^{-12}	2.20 10^{-12}	8.22 10^{-13}	2.37 10^{-13}	8.65 10^{-14}
1-butanol	87.62	74.1	2.53 10^{-12}	1.24 10^{-12}	3.47 10^{-13}	7.16 10^{-14}	
1-pentanol	104.42	88.2	1.75 10^{-12}	7.44 10^{-13}	1.84 10^{-13}		
1-hexanol	121.22	102.2	1.26 10^{-12}	4.94 10^{-13}	1.17 10^{-13}		
1-heptanol	138.03	116.2	9.44 10^{-13}	3.60 10^{-13}			
1-octanol	154.83	130.2	7.33 10^{-13}	2.61 10^{-13}			

Table S8. Diffusion coefficients (D_P) in EVOH for the homologeous row of 2-ketones.

	Molecular volume	Molecular weight	D_P				
	Å^2	g/mol	cm^2/s				
			80.4 °C	75.5 °C	70.6 °C	65.6 °C	60.6 °C
acetone	64.74	58.1	5.24 10^{-12}	2.22 10^{-12}	2.15 10^{-12}	7.61 10^{-13}	3.00 10^{-13}
2-butanone	81.54	72.1	3.93 10^{-12}	1.52 10^{-12}	1.03 10^{-12}	2.74 10^{-13}	5.26 10^{-14}
2-pentanone	98.34	86.1	2.63 10^{-12}	9.61 10^{-13}	4.49 10^{-13}	8.51 10^{-14}	
2-hexanone	115.15	100.2	1.93 10^{-12}	6.91 10^{-13}	2.37 10^{-13}		
2-heptanone	131.95	114.2	1.48 10^{-12}	5.09 10^{-13}	1.60 10^{-13}		
2-octanone	148.75	128.2	1.22 10^{-12}	4.01 10^{-13}			

Table S9. Diffusion coefficients (D_P) in EVOH for oxygen-containing heterocycles.

	Molecular volume	Molecular weight	D_P				
	Å^2	g/mol	cm^2/s				
			79.5 °C	74.6 °C	69.6 °C	64.6 °C	59.6 °C
furan	65.61	68.1	5.65 10^{-12}	4.48 10^{-12}	2.96 10^{-12}	1.47 10^{-12}	7.56 10^{-13}
2-methyl furan	82.17	82.1	4.89 10^{-12}	3.38 10^{-12}	1.79 10^{-12}	8.23 10^{-13}	2.28 10^{-13}
1,3-dioxolane	70.17	74.1	4.50 10^{-12}	2.76 10^{-12}	1.42 10^{-12}	6.86 10^{-13}	1.74 10^{-13}
tetrahydrofuran	77.98	72.1	3.13 10^{-12}	1.52 10^{-12}	6.23 10^{-13}	2.22 10^{-13}	
2-methyl-1,3-dioxolane	86.75	88.1	3.55 10^{-12}	1.81 10^{-12}	7.60 10^{-13}	2.85 10^{-13}	
2-ethylfuran	98.97	96.1	3.81 10^{-12}	2.02 10^{-12}	8.62 10^{-13}	3.79 10^{-13}	
1,4-dioxane	86.97	88.1	2.26 10^{-12}	1.03 10^{-12}	3.89 10^{-13}	1.29 10^{-13}	
furfuryl amine	93.70	97.1	5.15 10^{-13}	1.99 10^{-13}	3.42 10^{-13}		
furfuryl alcohol	90.43	98.1	3.88 10^{-12}	2.05 10^{-12}	7.10 10^{-13}	3.17 10^{-13}	

Table S10. Diffusion coefficients (D_P) in EVOH for aromatic substances.

	Molecular volume	Molecular weight	D_P		
	Å^2	g/mol	cm^2/s		
			80.4 °C	75.6 °C	70.5 °C
Benzene	84.04	78.1	3.82 10^{-12}	1.85 10^{-12}	8.02 10^{-13}
Toluene	100.60	92.1	3.01 10^{-12}	1.36 10^{-12}	5.25 10^{-13}
Ethyl benzene	117.41	106.2	2.05 10^{-12}	7.93 10^{-13}	2.46 10^{-13}
Propyl benzene	134.21	120.2	1.43 10^{-12}	6.64 10^{-13}	1.33 10^{-13}
Butyl benzene	151.01	134.2	1.13 10^{-12}	3.74 10^{-13}	
Naphthaline	128.03	128.2	1.04 10^{-12}	3.78 10^{-13}	
Methyl naphthaline	144.60	142.2	5.51 10^{-13}	1.78 10^{-13}	
Ethyl naphthaline	161.40	156.2	4.58 10^{-13}	1.33 10^{-13}	

Table S11. Diffusion coefficients (D_P) in EVOH for the homologeous row of formate esters determined from PE/EVOH/PE film.

	Molecular volume	Molecular weight	D_P			
	Å^2	g/mol	cm^2/s			
			80.2 °C	75.6 °C	70.6 °C	65.8 °C
methyl formate	57.16	60.1				2.31 10^{-12}
ethyl formate	73.97	74.1	4.69 10^{-12}	2.91 10^{-12}	1.50 10^{-12}	5.97 10^{-13}
propyl formate	90.77	88.1	3.10 10^{-12}	1.41 10^{-12}	5.43 10^{-13}	
butyl formate	107.57	102.1	2.13 10^{-12}	8.19 10^{-13}	2.79 10^{-13}	
pentyl formate	124.37	116.2	1.55 10^{-12}	5.12 10^{-13}	1.70 10^{-13}	
hexyl formate	141.17	130.2	1.15 10^{-12}	2.94 10^{-13}		
heptyl formate	157.98	144.2	9.29 10^{-13}	2.53 10^{-13}		

5. Determined activation energies for some of the measured permeants.

Table S12. Activation energies of diffusion E_A and pre-exponential factors D_0 determined within this study.

Substance	E_A	D_0	r^2	kinetic points	temperature range
	kJ/mol	cm^2/s			°C
acetone	135.0	$4.80\ 10^{8}$	0.9507	5	60.6 – 80.4
2-butanone	205.8	$1.20\ 10^{19}$	0.9605	5	60.6 – 80.4
2-pentanone	223.8	$3.31\ 10^{21}$	0.9756	4	65.6 – 80.4
methanol	41.8	$1.64\ 10^{-5}$	0.8837	5	61.3 – 81.3
ethanol	121.2	$6.20\ 10^{6}$	0.9658	5	61.3 – 81.3
1-propanol	197.6	$6.44\ 10^{17}$	0.9820	5	61.3 – 81.3
1-butanol	246.6	$7.12\ 10^{24}$	0.9590	4	66.9 – 81.3
furan	101.0	$6.11\ 10^{3}$	0.9700	5	59.6 – 79.5
2-methylfuran	148.4	$6.01\ 10^{0}$	0.9603	5	59.6 – 79.5
1,3-dioxolane	155.3	$5.53\ 10^{11}$	0.9660	5	59.6 – 79.5
tetrahydrofuran	176.1	$4.11\ 10^{14}$	0.9963	4	64.6 – 79.5
2-methyl-1,3-dioxolane	168.3	$3.17\ 10^{13}$	0.9978	4	64.6 – 79.5
2-ethylfuran	155.0	$3.67\ 10^{11}$	0.9978	4	64.6 – 79.5
1,4-dioxane	190.8	$4.35\ 10^{16}$	0.9968	4	64.6 – 79.5
Furfuryl alcohol	171.0	$8.66\ 10^{13}$	0.9934	4	64.6 – 79.5

6. Prediction of diffusion coefficients for *N*-(2-hydroxyethyl)maleimide and furfurylamine

Table S13. Prediction of Diffusion coefficients of furfurylamine and *N*-(2-hydroxyethyl)maleimide.

Structure	Molecular weight [g/mol]	Molecular volume [Å³]	Diffusion coefficient [cm^2/s] at 23 °C			
			LDPE	Adhesive	PET	EVOH
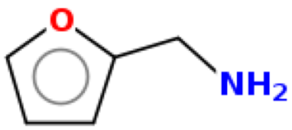	97.12	97.70	3.55 10^{-8} (worst case) from Piringer model with A_P' = 11.5, τ = 0 K, (Begley et al 2005)	3.55 10^{-8} (worst case) assumed as the same as for PE	1.86 10^{-15} (realistic) 1.60 10^{-14} (worst case) from Welle with a = 1.93·10^{-3} 1/K, b = 2.37·10^{-6} cm^2/s, c = 11.11·Å³ and d = 1.50·10^{-4} 1/K (Welle 2013)	9.26 10^{-19} (realistic) 5.21 10^{-17} (worst case) from Welle with a = 2.77·10^{-3} 1/K, b = 1.60·10^{-11} cm^2/s, c = 38.82 Å³ and d = 3.36·10^{-5} 1/K (this study)
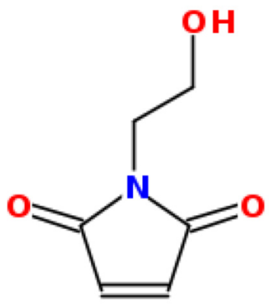	141.13	121.58	1.81 10^{-8} (worst case) from Piringer model with A_P' = 11.5, τ = 0 K, (Begley et al 2005)	1.81 10^{-8} (worst case) assumed as the same as for PE	2.25 10^{-16} (realistic) 1.94 10^{-15} (worst case) from Welle with a = 1.93·10^{-3} 1/K, b = 2.37·10^{-6} cm^2/s, c = 11.11 Å³ and d = 1.50·10^{-4} 1/K (Welle 2013)	1.79 10^{-20} (realistic) 1.00 10^{-18} (worst case) from Welle with a = 2.77·10^{-3} 1/K, b = 1.60·10^{-11} cm^2/s, c = 38.82 Å³ and d = 3.36·10^{-5} 1/K (this study)
